# Chloride Channelopathies of ClC-2

**DOI:** 10.3390/ijms15010218

**Published:** 2013-12-27

**Authors:** Miao Miao Bi, Sen Hong, Hong Yan Zhou, Hong Wei Wang, Li Na Wang, Ya Juan Zheng

**Affiliations:** 1 Department of Ophthalmology, the Second Hospital of Jilin University, Jilin University, Changchun 130041, Jilin, China; E-Mail: bimiaomiao1981@sohu.com; 2 Department of Ophthalmology, the China-Japan Union Hospital, Jilin University, Changchun 130033, Jilin, China; E-Mails: zhouhongyan7301@sina.com (H.Y.Z.); lnwang@jlu.edu.cn (L.N.W.); 3 Department of Colon and Anal Surgery, the First Hospital of Jilin University, Jilin University, Changchun 130021, Jilin, China; E-Mail: hongsen5413@sohu.com; 4 Department of Ophthalmology, the First Affiliated Hospital of Qiqihar Medical College, Qiqihar Medical University, Qiqihar 161006, Heilongjiang, China; E-Mail: wangyuting265@sohu.com

**Keywords:** ClCs, ClC-2, ClC-2 channels, ClC-2 chloride channelopathies

## Abstract

Chloride channels (ClCs) have gained worldwide interest because of their molecular diversity, widespread distribution in mammalian tissues and organs, and their link to various human diseases. Nine different ClCs have been molecularly identified and functionally characterized in mammals. ClC-2 is one of nine mammalian members of the ClC family. It possesses unique biophysical characteristics, pharmacological properties, and molecular features that distinguish it from other ClC family members. ClC-2 has wide organ/tissue distribution and is ubiquitously expressed. Published studies consistently point to a high degree of conservation of ClC-2 function and regulation across various species from nematodes to humans over vast evolutionary time spans. ClC-2 has been intensively and extensively studied over the past two decades, leading to the accumulation of a plethora of information to advance our understanding of its pathophysiological functions; however, many controversies still exist. It is necessary to analyze the research findings, and integrate different views to have a better understanding of ClC-2. This review focuses on ClC-2 only, providing an analytical overview of the available literature. Nearly every aspect of ClC-2 is discussed in the review: molecular features, biophysical characteristics, pharmacological properties, cellular function, regulation of expression and function, and channelopathies.

## 1. Introduction

Before the late 1990s, chloride channels (ClCs) were not studied in depth because it was thought that these channels were in electrochemical equilibrium across cell membranes. In the decade between 1993 and 2003, ClCs began to gain increasing attention owing to the findings that ClCs are linked to human disease and the realization that Cl^−^ is actively transported. Indeed, we now know that ClCs are active out of electrochemical equilibrium and are involved in a variety of cellular functions.

ClC proteins are encoded by genes of the ClC family and are expressed in virtually all phyla. Thus far, nine different genes of the ClC family (CLCN) have been molecularly identified in mammals. Molecularly, the ClC family can be further divided into three distinct subfamilies: ClC-1, -2, -Ka/K1, and -Kb/K2; ClC-3, -4, and -5; and ClC-6 and -7. Functionally, ClCs can be categorized into two functional groups: voltage-gated chloride channels and Cl^−^/H^+^ exchangers, with the former including four proteins: ClC-1, -2, -Ka/K1, and -Kb/K2. The functional unit of ClCs is a homodimer [1–3]. ClCs predominantly carry Cl^−^ flux across the plasma membrane and intracellular membranes in most cell types, though they exhibit low selectivity among anions [3,4]. ClCs are believed to participate in maintenance of the resting membrane potential, cell volume regulation, and acidification of intracellular compartments such as endosomes and lysosomes [1–3].

ClC-2 is one of the nine mammalian members of the ClC family; it was originally cloned from rat heart and brain [5] and subsequently from rabbit heart [6]. ClC-2 is a two-pore homodimeric, voltage-gated Cl^−^ channel [7–9]. It possesses unique biophysical characteristics, pharmacological properties, and molecular features that confer its unique cellular functions and distinguish it from other ion channels, including other Cl^−^ channels. Northern blot analysis indicated that ClC-2 has a wide organ distribution and is ubiquitously expressed. Published studies consistently point to a high degree of conservation of ClC-2 function and regulation across various species from nematodes to humans over vast evolutionary time spans [10]. Because of these properties, ClC-2 has been intensively and extensively studied over the past two decades. A plethora of information has accumulated to advance our understanding of the pathophysiological functions of ClC-2, but many controversies still exist. Therefore, it is necessary to sort out the information, analyze the research findings, and integrate different views to have a better understanding of ClC-2. Though several excellent review articles on ClCs have been published [4,11–14], there has not been one focusing on ClC-2 during the last decade. The present review article aims to provide an analytical overview of the available literature data on ClC-2. The content of this essay includes molecular features, biophysical characteristics, pharmacological properties, cellular function, regulation of expression and function, and channelopathies, with final concluding remarks and future perspectives.

## 2. Biophysical Properties of ClC-2

The biophysical characteristics of an ion channel current are the phenotypes reflecting the molecular features and conferring the cellular functions of this channel. ClC-2 channel dimers exhibit two largely independent protopores that are opened and closed individually as well as by a common gating process. The current carried by ClC-2 channels has been well characterized electrophysiologically in both native cells and heterologous systems expressing cloned CLCN2 gene. ClC-2 channel current possesses unique biophysical characteristics distinct from other Cl^−^ channels and transporters.

**Figure 1.**
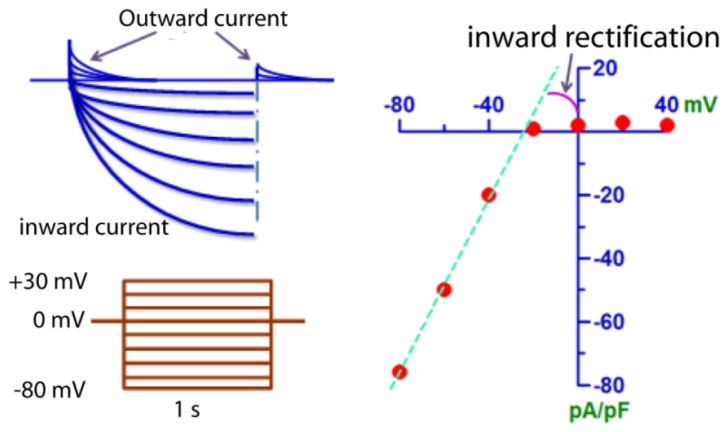
Biophysical properties of ClC-2 channels in mouse cardiomyocytes. (**Left**) A typical example of the currents mediated by ClC-2 channels elicited by the voltage protocols shown in the inset. Cells were voltage clamped by stepping from a holding potential of 0 mV to various potentials between −80 and 30 mV in 10 mV increments for 1 s, following by 400 ms steps to 0 mV. The pipette solution contained 120 mM *N*-methyl-d-glucamine-Cl, 5 mM MgATP, 0.1 mM NaGTP, 5 mM EGTA, 5 mM HEPES; pH 7.4, and 300 mOsm. The bath solution contained 102 mM NaCl, 1 mM MgCl_2_, 1 mM CaCl_2_, 2 mM BaCl_2_, 10 mM CsCl, 10 mM HEPES, 10 mM glucose; pH 7.4, and 300 mOsm. Hyperpolarization elicits Cl^−^ efflux to mediate large inward currents; in contrast, depolarizing pulses provoke Cl^−^ influx to mediate small outward currents. (**Right**) The current-voltage (I–V) relationship of ClC-2 currents. Deviation of actual currents from the theoretical linear regression line indicates the inward rectifying property of ClC-2 channels.

Perhaps, the physiological counterpart of the CLCN2 gene has been best described in cardiac myocytes by Duan *et al.* [15]. ClC-2 is largely closed under resting conditions, but activated by membrane hyperpolarization (−40 to −140 mV) relative to the equilibrium potential of Cl^−^ (−30 mV); and under isotonic conditions, the activation time course is slow (Figure 1). When activated, it carries inward current by Cl^−^ outflow with a strong inwardly rectifying property. The inward rectifying property of ClC-2 is interesting, as in cardiac myocytes, the well-characterized inward rectifying currents are mediated by cationic ion channels such as Kir and hyperpolarization-activated *I*_f_ channels. ClC-2 represents the only anionic inward rectifier in heart identified thus far.

The ClC-2 current is highly sensitive to changes in cell volume. Under hypotonic conditions, cell swelling accelerates the activation kinetics and increases the current amplitude; in contrast, hypertonic cell shrinkage inhibits the current [5,15,16].

ClC-2 channel activity is also modulated by changes in extracellular pH. The activity of ClC-2 has a biphasic response to extracellular pH with activation by moderate acidification followed by abrupt channel closure at pH values less than approximately pH 7 [17].

The ClC-2 channel has a unitary conductance of 2–3 pS [18–20]. ClC-2-ClC-2 concatemers expressed in Xenopus oocytes exhibited pores with a single channel conductance of approximately 3 pS [18]. Similarly, ClC-2 channels heterologously expressed in mammalian cell lines have a small single channel conductance of 2–3 pS [19,20]. In addition, ClC-2 channels demonstrate a halide anion permeability sequence of Cl^−^ ≥ Br^−^ >> I^−^ ≥ F^−^ and markedly greater selectivity over organic anions such as Asp^−^ [6,15,21,22].

## 3. Pharmacological Properties of ClLC-2

The pharmacological properties of an ion channel current also reflect the molecular structural features of the channel protein and provide unique opportunities and tools for basic research as well as potential clinical applications for disease therapy. ClC-2 has been extensively investigated in terms of its pharmacological sensitivity to a number of research agents and therapeutic drugs, and the findings have not only helped us to understand ClC-2 physiology but also have assisted in the development of ClC-2-specific blockers.

### 3.1. ClC-2 Inhibitors

The ClC-2 channel was found to be blocked by carboxylic acid derivatives (such as diphenylamine-2-carboxylic acid, DPC), 5-nitro-2-(3-phenylpropylamino)benzoic acid (NPPB), and Cd^2+^ but is largely unaffected by disulfonic stilbene derivatives (such as SITS) and tamoxifen (TMX) [6,15].

Aside from these compounds, a study identified *Leiurus quinquestriatus hebraeus* venom as containing a peptide toxin inhibitor of ClC-2 channels [23]. This toxin, named GaTx2, was found to inhibit ClC-2 channels with a voltage-dependent apparent Kd value of approximately 20 pM, making it the highest affinity inhibitor of any chloride channel. GaTx2 slows ClC-2 activation but does not block open channels. GaTx2 does not affect any other chloride channel and may be considered a ClC-2-specific inhibitor. The high affinity and specificity to ClC-2 channels displayed by GaTx2 should make it a very powerful pharmacological tool to probe the structure and function of ClC-2.

### 3.2. ClC-2 Activators

In addition to inhibitors, ClC-2 channels can also be activated by pharmacological agents. One such example is acid-activated omeprazole [24]. Omeprazole is an anti-ulcer agent that, when activated by acid, forms a charged species that reacts with cysteines on proteins [25,26]. The ClC-2 channel protein carries such cysteine residues, which may be accessible to react with acid-activated omeprazole [24].

Perhaps the most striking application of ClC-2 pharmacology is lubiprostone (Amitiza), a ClC-2 activator belonging to a new class of compounds known as prostones. Lubiprostone is a bicyclic fatty acid that can act on ClC-2 channels in the apical membrane of intestinal epithelial cells. Preclinical trials have shown a high specificity of the drug for ClC-2 channels. Activation of ClC-2 channels by lubiprostone causes Cl^−^ secretion to induce the passive movement of sodium and water into the intestinal lumen, leading to a net increase in isotonic fluid to improve bowel function. In several double-blind, placebo-controlled clinical trials, lubiprostone was shown to increase the number of spontaneous bowel movements, stool consistency, bloating, and global assessment of constipation, compared with placebo [27–29]. Because of the solid clinical outcomes and relatively clear mechanisms of action, the US Federal Drug Administration (FDA) has approved lubiprostone for the treatment of chronic constipation and the treatment of women with irritable bowel syndrome with constipation.

## 4. Molecular Features of ClC-2

### 4.1. Basic Protein Structure

**Figure 2.**
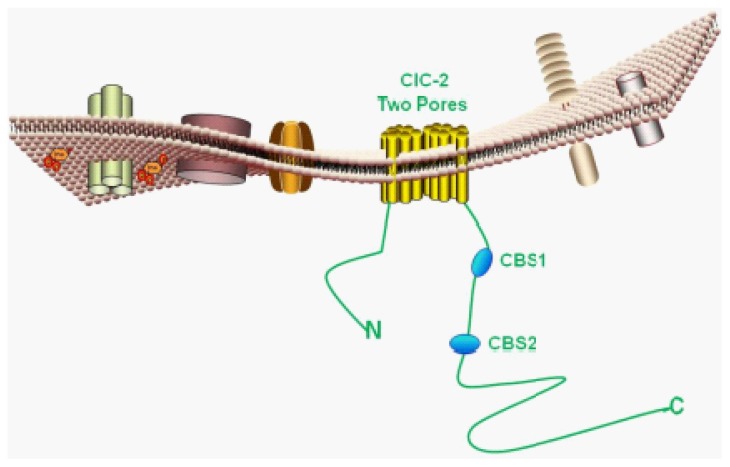
Molecular/structural features of ClC-2 channels. The predicted membrane topology of a ClC-2 monomer is shown.

The CLCN2 gene encodes the ClC-2 protein, consisting of 907 amino acids with a molecular mass of 99 kDa, which shares approximately 50% homology with ClC-0 and ClC-1 channel proteins. After the cloning of CC-0 in 1996, the functional structure of this channel was unambiguously demonstrated to be homodimeric, with each protopore contributing to a chloride conduction pathway [7,30]. Single channel recording of ClC2 suggests a similar dimeric structure for ClC-2 [18–20]. In 2002, Dutzler *et al.* revealed a 3.0 Å resolution bacterial ClC structure, confirming that ClC proteins formed homodimeric functional units [31]. Thus, ClC-2 is a two-pore homodimeric channel (Figure 2).

### 4.2. Structure-Function Relationships

Structure-function relationships of ClC-2 channel proteins have been investigated by several groups [21,32–35]. Crystallographic and functional studies of other ClCs suggest that a conserved glutamate residue carboxylate side-chain can close protopores by interacting with Cl^−^. Niemeyer *et al.* [32] revealed that ClC-2 gating depends on intracellular but not extracellular Cl^−^ and that neutralization of E217, the homologous pore glutamate, leads to loss of sensitivity to intracellular Cl^−^ and voltage. E217 is a hyperpolarization-dependent protopore gate in ClC-2, and access of intracellular Cl^−^ to a site normally occupied by its side-chain in the pore stabilizes the open state.

**Figure 3.**
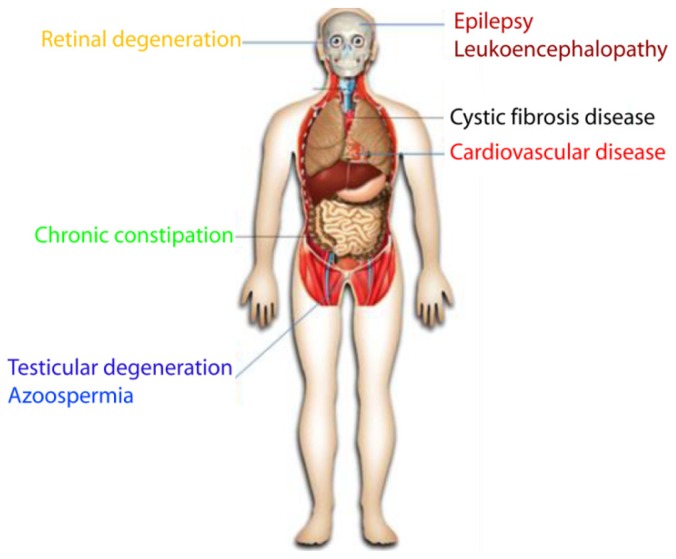
ClC-2 chloride channelopathies. Schematic illustration of human diseases that can be caused by loss of ClC-2 channels.

The CBS domain is a conserved sequence region named after cystathionine beta synthase. The CBS domain is composed of a beta-alpha-beta-beta-alpha secondary structure pattern that is folded into a globular tertiary structure containing a three-stranded antiparallel β-sheet with two α-helices on one side. These structures contain a conserved pair of tightly interacting CBS motifs connected by a variable linker and followed by an unstructured distal end. The CBS1–CBS2 linker consists of a flexible region for regulation and an α-helical region preceding CBS2 (Figure 3). Tight binding of CBS1 and CBS2 is a general feature of ClC carboxy-termini. Garcia-Olivares *et al.* [36] investigated the role of the *C*-terminal domain in modulating fast and slow gating of human ClC-2 channels. Their results showed that partial truncation of the *C*-terminal fragment distal to CBS2 leads to an incomplete CBS1–CBS2 pair and abolishes the function of ClC-2 by locking the channel in a closed position; whereas, unexpectedly, complete removal of the *C*-terminus preserves function of ClC-2. ClC-2 channels without the *C*-terminus exhibit fast and slow gates that activate and deactivate significantly faster than in wild-type channels. These results indicate that the C-terminus is not strictly required for slow gating and that the cooperative gating resides in other regions of the channel protein.

It has been mentioned earlier that ClC-2 channel activity is critically modulated by changes in extracellular pH. With the use of site-directed mutagenesis, the extracellular region EELE (amino acids 416–419) was identified as the pH sensor and the amino acid Glu-419 was found to play the key or predominant role in activation of the ClC-2G Cl- channel by extracellular acid [37]. Additionally, a separate group [33] described that neutralization of Lys566 at the end of the transmembrane domains results in the disappearance of inward rectification, the appearance of outward rectification, and a shift in voltage dependence, but it leaves the basic gating mechanism, including swelling activation, intact. In contrast, mutations in the cytoplasmic loop between transmembrane domains D7 and D8 abolish the activation process by constitutively opening the channel without changing its pore properties.

### 4.3. Promoter Region

Using RNA primer extension techniques, Cid *et al.* [10] identified that the major transcription start site of the ClC-2 gene is localized 100 bp upstream of the putative translation initiation codon. Analysis of the 5′-flanking sequence further revealed that the 5′-flanking region for transcriptional regulation of this gene contains a high GC content and lacks common transcriptional elements such as TATA and CCAAT boxes [10]. However, in the study reported by Chu *et al.* [38], three CCAAT boxes within the 1,930-bp region upstream of the ClC-2 gene were identified, one of which is close to the ClC-2 coding sequence (−738 bp), and the other two are in the middle (−1101 and −1176 bp, respectively) [38]. Multiple GC boxes were also identified in this region. Four of them are within a 391-bp region upstream from the first Met codon of ClC-2: the first three GC boxes are highly conserved in human ClC-2 as reported by Cid *et al.* [10]. In addition, there are two potential stem-loop structures in this region, with the first overlapping with the first two GC boxes and the second stem-loop structure residing between the third and fourth GC boxes. Consistent with the observation that promoters with GC-rich regions are usually without TATA promoters, no TATA box was found in this 1930-bp intervening region.

These molecular features form the basis for the cellular functions and define the phenotypic biophysical and pharmacological properties of ClC-2 channels. As will be introduced in the appropriate sections, mutations or functional modification of the molecular/structural features of ClC-2 not only can correspondingly alter the electrophysiological properties and cellular functions, but they also can cause diseases.

## 5. Cellular Function of ClC-2

We have to admit that at this time the physiological role of ClC-2 channels remains incompletely understood and even uncertain. A part of the reason for this is because most studies have been carried out on recombinant ClC-2 channels in heterologous expression systems. Another reason may be that ClC-2-deficient mice were found to lack overt abnormalities except for severe degeneration of the retina and the testes, contrary to expectations for ClC-2 involvement in many important functions [39]. On the basis of the biophysical characteristics and spatial as well as temporal expression patterns, several cellular functions have been proposed and also supported by some experimental evidence.

### 5.1. Regulation of Cell Volume

Volume regulatory mechanisms are critical to maintain structural integrity and proper cellular functions of living cells. It is not surprising that the ClC-2 channel has been generally considered a regulator of cell volume because of its intrinsic volume sensitivity [40]. In cardiac myocytes, several mechanisms operate to precisely maintain their volume/size in the face of osmotic perturbations [41]. However, while the impact of osmolarity on ClC-2 function has been investigated in detail, solid experimental evidence for the role of ClC-2 in regulating cell volume is presently lacking. On the contrary, some negative data have been published. In their study to examine the role of ClC-2 in salivary gland function, Nehrke *et al.* [42] found that the magnitude and biophysical characteristics of the volume- and calcium-activated Cl^−^ currents in these cells are unaffected by the absence of ClC-2 in ClC-2 knockout mice. Although ClC-2 appears to contribute to fluid secretion in some cell types, both the initial and sustained salivary flow rates are normal in the knockouts following *in vivo* stimulation with pilocarpine, a cholinergic agonist. In addition, the electrolytes and protein contents of the mature secretions are normal. Further, parotid acinar cells from ClC-2-deficient mice recover volume with similar efficiency as their wild-type littermates [42]. The role of ClC-2 in volume regulation may depend on its expression level relative to other swelling-activated chloride channels.

### 5.2. Regulation of Transepithelial Transport

Perhaps, the ClC-2 knockout animal model has provided the most convincing evidence for the cellular functions of this channel. ClC-2-deficient mice were first established by Bösl *et al.* [39]. This model exhibits that the disruption of ClC-2 has a dramatic impact on two organs whose interior is protected by a blood–organ barrier: the seminiferous tubule of the testis and the retina. ClC-2 disruption entails the death of two cell types, which depend on supporting cells that form the blood–testes and blood–retina barriers. It seems that the function of ClC-2 is most critical for cells that depend on a closely associated and actively transporting epithelium for their function and survival. The authors accordingly proposed that ClC-2 is crucial for controlling the ionic environment of these cells and that the ClC-2 channel plays an important role in the transepithelial transport of Sertoli cells to maintain ionic homeostasis in seminiferous tubules [39].

### 5.3. Regulation of Tight Junction Function

A cellular function of ClC-2 closely related to the aspects introduced above is the regulation of tight junction function. There is evidence for an important role of ClC-2 in the recovery of epithelial barrier function by orchestrating repair of apical tight junctions [43–45]. The tight junctions are formed by a blend of transmembrane proteins (e.g., occludin and claudins) linked by cytoplasmic plaque proteins to the cytoskeleton. In the intestine, the epithelium regulates water, nutrient, and ion transport while providing a barrier against toxins and pathogenic organisms. The apical intercellular tight junctions are largely responsible for barrier function, and loss of intestinal barrier function contributes to a number of critically important intestinal diseases such as inflammatory bowel disease, celiac disease, and ischemia-reperfusion injury [46]. It has been demonstrated that intestinal epithelial barrier function is altered and barrier recovery is impaired in the absence of ClC-2, references [45,47,48] explored the link between ClC-2 and apical tight junctions. Their results revealed that ClC-2 plays an important role in epithelial barrier development as well as maintenance and that ClC-2 mediates barrier function via membrane targeting of the tight junction protein occludin. Knockdown of ClC-2 by an RNAi approach significantly delayed barrier development during monolayer formation associated with disruption of occludin localization at the tight junctions. The absence of ClC-2 during monolayer formation led to subapical, diffuse localization of occludin instead of membrane localization. An earlier study by Moeser *et al.* [44] demonstrated that application of the ClC-2 activator lubiprostone to ischemia-injured mucosa induces concentration-dependent increases in transepithelial electrical resistance, reduces mucosal-to-serosal fluxes of ^3^H-labeled mannitol, and restores occludin localization to tight junctions.

### 5.4. Regulation of Pacemaker Activity

Sino-atrial node (SAN) cells are known to be the site where the primary excitation or pacemaker activity in the heart originates [49]. The generation of pacemaker activity is primarily due to spontaneous diastolic depolarization, which is governed by several ion channel currents including hyperpolarization-activated nonselective cationic “funny” current (*I*_f_) and T-type and L-type Ca^2+^ currents, and is regulated by a wide spectrum of factors [50–52]. Under physiological conditions, the Cl^−^ equilibrium potential is approximately −30 mV, which is about 30 mV more positive than the normal resting membrane potential (−60 mV) in SAN cells [53]. At membrane potentials more negative than −30 mV, ClC-2 channels carry Cl^−^ efflux to generate inward current because of their inwardly rectifying properties [15,41]. This property is expected to contribute to the regulation of resting membrane potential and pacemaker activity.

It was shown that in isolated guinea-pig SAN cells, hypotonic stress increases the diastolic depolarization slope and decreases the maximum diastolic potential, action potential amplitude, and therefore the rhythm of pacemaker activity [54]. Coincidently, Cl^−^ current was found to be activated by hyperpolarization and hypotonic cell swelling [54]. Moreover, the presence of ClC-2 transcripts and proteins in guinea-pig SAN cells was confirmed by RT-PCR and immunohistological analyses. Most notably, the pacemaker-promoting effect of hypotonic stress was essentially reversed by intracellular dialysis of the anti-ClC-2 antibody. Furthermore, telemetry electrocardiograph studies in conscious homozygous ClC-2 knockout mice revealed a decreased chronotropic response to acute exercise stress when compared to their age-matched wild-type and heterozygous littermates. It should be noted that in this study [54], targeted inactivation of ClC-2 did not alter the intrinsic heart rate. These results provide compelling evidence for the critical role of ClC-2 channels in the regulation of cardiac pacemaker activity, which is manifested under stressed or pathological conditions [54].

### 5.5. Regulation of Vascular Smooth Muscle Cell (VSMC) Proliferation

Abnormal proliferation and directed migration of VSMCs are important steps in the pathogenesis of atherosclerosis and intimal hyperplasia [55]. Insulin-like growth factor (IGF)-I, as an active mitogen and strong chemo-attractant, has been documented to induce proliferation and migration of VSMCs [56,57]. Intriguingly, IGF-I-induced VSMC proliferation was found to be suppressed by the Cl^−^ channel blockers NPPB and IAA94, but not by DIDS, and it was accompanied by upregulated expression of ClC-2 mRNA and protein [58]. Inhibitors of PI3-kinase, a downstream mediator of the IGF-I signaling pathway, attenuated the IGF-I-upregulated ClC-2 expression and cell proliferation. More direct evidence for the involvement of ClC-2 is was shown by the ability of ClC-2 siRNA to abolish IGF-I-induced cell proliferation [58].

### 5.6. Regulation of Glioma Cell Proliferation

Consistent with the cell proliferation-inhibitory effects of ClC-2 in VSMCs, ClC-2 has also been shown to suppress tumor cell growth. It has been reported that knockdown of ClC-2 by its siRNAs reduces the cell growth rate of human U-87 malignant glioma cells, arrests the cell cycle in the G1 phase, and suppresses the formation of cell colonies [59].

### 5.7. Regulation of Neuronal Excitability

It is known that synaptic inhibition by GABA_A_ receptors requires a transmembrane Cl^−^ gradient; that is, the electrochemical potential for Cl^−^ determines whether GABA_A_- or glycine-receptor Cl^−^ channels are inhibitory or excitatory. Activation of ClC-2, an inward-rectifier that helps extrude Cl^−^, may prevent a rise of intracellular Cl^−^ above equilibrium, ensuring an inhibitory response. Staley and colleagues [60,61] linked the expression of ClC-2 and the associated inward currents to an inhibitory response to GABA. Expression of ClC-2 in dorsal root ganglia was also shown to convert the response to GABA from excitatory to inhibitory [62]. In addition, ClC-2 has been shown to constitute background conductance and mediate an efflux pathway for chloride in CA1 pyramidal cells [63]. Loss of ClC-2 leads to hyperexcitability in CA1 pyramidal cells, which is balanced by increased inhibition caused by hyperexcitability of inhibitory interneurons, thus preventing epilepsy in ClC-2 knockout mice [63]. Furthermore, ClC-2 has been reported to selectively regulate fast-spiking parvalbumin-expressing basket cell synapses in the hypocampus [64]. Functional studies using a dynamic clamp to insert virtual ClC-2 channels into rat CA1 pyramidal cells with and without native ClC-2 channel blockade also confirmed that ClC-2 reduces spiking independently of inhibitory synaptic transmission, highlighting the importance of ClC-2 in regulating neuronal activities [65]. However, as is discussed below, the role of ClC-2 in regulating the intracellular Cl^−^ concentration in neurons is not supported by knockout experiments.

### 5.8. Important Notes to Consider

It should be noted that several anticipated functions of ClC-2 are not supported by the observations obtained from ClC-2 knockout animals [39]. First, ClC-2 is expressed in the apical membranes of lung epithelia and in the fetal lung, and thus it has been postulated to play a role in Cl^−^ and fluid secretion and lung development, as lung development depends on fluid secretion [66,67]. However, ClC-2 knockout mice develop perfectly normal lungs. Second, the normal kidney development of ClC-2 knockout mice indicates that ClC-2 is not required in this process [68]. Third, normal gastric acidification disproves the previous notion that proton secretion in the stomach is rate-limiting [69]. Finally, if ClC-2 is indeed critical for regulation of the intracellular Cl^−^ gradient, then a loss of ClC-2 function should cause an extensive excitatory GABA response leading to seizures. Intriguingly, a genome search for susceptibility loci of common idiopathic generalized epilepsy identified a locus at 3q26 [70] in the vicinity of the human CLCN2 gene [71]. However, in the study reported by Bösl *et al.* [39], no spontaneous seizures were observed in ClC-2 knockouts, and the threshold to a seizure-inducing agent was not affected significantly. This finding seems to rule out a widespread, dominant role of ClC-2 in the regulation of neuronal intracellular Cl^−^. Overall, ClC-2-deficient mice lack overt abnormalities except for severe degeneration of the retina and the testes, which led to selective male infertility [39].

## 6. Regulation of ClC-2 Expression and Function

We now know that ClC-2 channel activities and thus its cellular functions are dynamically and cooperatively regulated by a wide array of extracellular, transmembrane, and intracellular factors. The regulation occurs at both the expression and functional levels, affecting the levels of ClC-2 mRNA and/or protein as well as ClC-2 channel activities. The physiological contexts under which such regulation occurs and the molecular mechanisms that mediate regulation are not yet completely understood, though we have been able to gain certain insight after years of research.

### 6.1. Expression Regulation

#### 6.1.1. Transcriptional Regulation

In rats, lung ClC-2 expression is rapidly downregulated at birth. This downregulation occurs when the lung switches from Cl^−^ and fluid secretion to net fluid absorption, an important step for preparing the fetal lung for air breathing. Yet the mechanisms remained unclear. Reference [38] conducted a study to characterize the ClC-2 promoter, define key elements in the promoter, and elucidate the transcriptional regulatory mechanisms. They found that the ClC-2 promoter is GC rich and lacks a TATA box. By construction of a series of promoter-luciferase constructs, a 67-bp GC box-containing sequence in the promoter was shown to be critical to ClC-2 expression in primary and immortalized fetal lung epithelial cells. Electrophoretic mobility shift assays with antibody supershifts demonstrated that the Sp1 and Sp3 transcription factors are expressed in fetal lung nuclei and interact with the GC box sequences in the ClC-2 promoter. Immunoblotting analysis showed that Sp1 and Sp3 are perinatally downregulated in the lung along with ClC-2 downregulation. This work suggests that Sp1 and Sp3 activate CLCN2 gene transcription and that reduction in Sp1 and Sp3 at birth explains perinatal downregulation of ClC-2 in the lung.

In line with these results, Holmes *et al.* [72] have also identified consensus binding sites for the transcription factors Sp1 and Sp3 within the first 237 bp in the ClC-2 promoter region. These authors then demonstrated that transient overexpression of either Sp1 or Sp3 induces ClC-2 protein in adult rat Type II cells that have low levels of endogenous ClC-2. Furthermore, application of a competitive inhibitor of Sp1 and Sp3 reduced ClC-2 expression in fetal rat Type II cells that express abundant endogenous ClC-2.

Further studies suggest that glycosylation of Sp1 is crucial to expression regulation of ClC-2. ClC-2 is highly expressed in mammalian fetal airway epithelia during the period of maximal fluid secretion. A high level of luminal ClC-2 protein expression is maintained by Sp1 until birth. Using fetal (preII-19) and adult (L2) rat lung Type 2 cell lines, Vij *et al.* [73] demonstrated that the phosphorylated and glycosylated form of Sp1 is the active form for its transactivation of ClC-2 expression. Exposure of either cell line to high-dose glutamine to induce glycosylation of Sp1 promoted ClC-2 expression, whereas exposure to tunicamycin to inhibit Sp1 glycosylation reduced ClC-2 expression. *In vivo* ClC-2 expression is similarly regulated: Sp1 from a 6-week-old murine lung is hyperphosphorylated and hyperglycosylated along with high ClC-2 expression, compared with Sp1 from a 16-wk-old lung with low ClC-2 expression.

The ClC-2 DNA sequence contains a consensus site (Ser82) for phosphorylation by the serum and glucocorticoid inducible kinase isoforms SGK1-3 (belonging to a serine/threonine-protein kinase subfamily) [74]. ClC-2 expression in *Xenopus* oocytes induces inwardly rectifying currents that increased upon coexpression of SGK1-3 and protein kinase B (PKB). Intriguingly, disruption of the SGK phosphorylation site fails to affect the stimulatory effect on ClC-2 channel activities. SGKs can phosphorylate the ubiquitin ligase Nedd4-2 and prevent Nedd4-2 from binding to its target. ClC-2 activity decreases upon Nedd4-2 coexpression, an effect reversed by the kinases. Chemiluminescence experiments showed that ClC-2 membrane abundance is enhanced by SGKs and diminished by Nedd4-2. Evidently, SGK1-3 and Nedd4-2 regulate ClC-2 at least in part by modulating ClC-2 abundance at the plasma membrane [74].

#### 6.1.2. Transcript Stabilization

Evidence exists that expression of ClC-2 is not only regulated at the transcriptional level but also at the post-transcriptional level. The study by Blaisdell *et al.* [75] revealed that ClC-2 mRNA stability declines over time. Keratinocyte growth factor (KGF) is a mitogenic factor in epithelial cells, which can induce cystic dilation of fetal lung explants. Coincidently, ClC-2 was found to be highly expressed on the apical surface of the respiratory epithelium but markedly downregulated after birth. Blaisdell *et al.* [75] investigated the relationship between KGF regulation and CLC-2 expression in the fetal lung. They demonstrated that KGF treatment upregulates CLC-2 mRNA and protein levels in primary fetal rat distal lung epithelial cell monolayers. Promoter-reporter gene experiments revealed that KGF does not act by stimulating CLCN2 transcription, but by stabilizing CLCN2 mRNA, as inhibition of new mRNA synthesis with actinomycin D does not affect the effects of KGF. In this way, KGF may positively influence pulmonary chloride and fluid secretion by a secondary pathway affecting CLC-2 degradation. Interferon-gamma (IFN-γ), a cytokine crucial for immunity against intracellular pathogens, has also been reported to regulate ClC-2 mRNA stability. IFN-γ is known to regulate Cl^−^ channels and fluid transport in the lung. The study by Chu *et al.* [76] linked this regulation to ClC-2. They found that IFN-γ increases ClC-2 transcripts in the human sub-bronchial gland cell line Calu-3 cells. Reporter gene assays with a minimal promoter showed that IFN-γ does not activate the promoter; instead, IFN-γ significantly increases ClC-2 transcript stability [76].

#### 6.1.3. Ubiquitination

Ubiquitination is a signal for proteasomal degradation, protein endocytosis, intranuclear trafficking, viral budding, and endosome trafficking [77]. It has been noticed that Cl^−^ channel activity varies during the cell cycle, so it is thought to play a role in cell cycle progression as well as membrane potential and cell volume regulation [78,79]. Immunoblot and immunocytochemical analyses revealed that ClC-2 channel protein is expressed predominantly at the M phase, whereas RNA blot analysis showed that ClC-2 mRNA is not altered during the cell cycle, using cells that were cycle-synchronized by serum depletion/replenishment [80]. The ClC-2 channel was identified as a target of regulation by the M phase-specific cyclin-dependent kinase p34cdc2/cyclin B; the *C* terminus of ClC-2 is directly phosphorylated by p34cdc2/cyclin B. As a result, ClC-2 channel activities are inhibited by p34cdc2/cyclin B in *Xenopus* oocytes with ClC-2 overexpression [80]. This finding is supported by the fact that expression of ClC-2 channel proteins is M phase-dependent with the highest expression in dividing cells at the M phase [80]. Coincidently, the ClC-2 channel protein is ubiquitinated at the M phase, which is controlled by p34cdc2/cyclin B phosphorylation of the channel, as the ubiquitination is suppressed by incubation with the p34cdc2/cyclin B inhibitor and nearly abolished in ClC-2 channels with S632A mutation (the p34cdc2/cyclin B phosphorylation site). The M phase-specific expression and phosphorylation of the ClC-2 channel protein suggest a physiological role of ClC-2 channels in the cell cycle.

#### 6.1.4. Trafficking Regulation

Another mechanism for post-transcriptional regulation of ClC-2 is trafficking. [81] found that ClC-2 is internalized via dynamin-dependent endocytosis; the change of ClC-2 proteins in surface expression evoked by ATP depletion is partially mimicked by inhibition of dynamin function using a dynamin dominant-negative mutant (DynK44A). Trafficking via the early endosomal compartment occurs in part through rab5-associated vesicles, and recycling of ClC-2 to the cell surface occurs through a rab11-dependent pathway. Thus, it appears that the internalization of ClC-2 by endocytosis is inhibited by metabolic stress, highlighting the importance for understanding the molecular mechanisms mediating the endosomal trafficking of this channel. A study by Cornejo *et al.* [82] confirmed the trafficking regulation of ClC-2 channels. They demonstrated that internalization and recycling of ClC-2 channels is kinetically rapid: approximately 70% is recycled after a 4–8-min internalization. The internalization by endocytosis of ClC-2 is determined by the tyrosine 179 (Y179) located within an endocytic motif. Rapid recycling accompanied by an even faster internalization account for the abundant presence of ClC-2 in intracellular membranous structures.

In addition to regulation by the internalization/recycling mechanism, ClC-2 trafficking is controlled by protein insertion as attested recently by the study documented by Hosseinzadeh *et al.* [83]. This regulation is performed by Janus kinase-2 (JAK2), a nonreceptor tyrosine kinase. JAK2 is activated by cell shrinkage and may thus participate in cell volume regulation. The ClC-2 current elicited in *Xenopus* oocytes with transient overexpression of ClC-2 was significantly decreased following coexpression of JAK2, and the suppression could be prevented by the JAK2 inhibitor AG490. Chemiluminescence analysis revealed that JAK2 decreases ClC-2 channel protein abundance in the cell membrane. Inhibition of channel protein insertion by brefeldin A gives rise to identical effects to JAK2. These results indicate that JAK2 might slow channel protein insertion rather than accelerate channel protein retrieval from the cell membrane.

#### 6.1.5. Regulation by Hormones

##### 6.1.5.1. Thyroid Hormone

Santos *et al.* [84] studied the effect of thyroid hormones on ClC-2 expression in the rat kidney by using hypothyroid rats with or without thyroxine (T4) replacement and hyperthyroid rats. Renal ClC-2 expression was found to be decreased in hypothyroid rats and increased in hyperthyroid rats. In addition, semi-quantitative RT-PCR of different nephron segments showed that these changes are due exclusively to the modulation of ClC-2 mRNA expression by thyroid hormone in convoluted and straight proximal tubules. Furthermore, ClC-2 expression at both the mRNA and protein levels was increased by T4 in a dose-dependent fashion.

##### 6.1.5.2. Aldosterone

It is well known that Na^+^ reabsorption in the kidney is regulated by aldosterone. It was shown that a high-Na^+^ diet reduces renal mRNA and protein levels of ClC-2 [85]. In addition, an adrenalectomy downregulates renal expression of ClC-2 mRNA, which is effectively restored by plasma aldosterone replacement. Detailed analysis of different segments of the nephron indicate that these changes are likely to be secondary to the modulation of ClC-2 mRNA expression by aldosterone in the cortical and medullary segments of the thick ascending limbs of Henle’s loop.

##### 6.1.5.3. Estrogen

Nascimento *et al.* demonstrated that ClC-2 is significantly downregulated at both the mRNA and protein levels in rats subjected to an ovariectomy, and these changes are restored to control levels after treatment with low doses of estradiol [86]. A higher dose of estradiol leads to an even greater increase in ClC-2 expression, and this change appears to be caused by the modulation of ClC-2 mRNA expression in the proximal tubule.

#### 6.1.6. Alpha1-Adrenoceptors

Acute sympathetic denervation of the small intestine upregulates α1-adrenoceptors on villus enterocytes, and activation of these α1-adrenoceptors inhibits Cl^−^ secretion. α1-Adrenoceptor activation significantly decreases ClC-2 protein levels in both the villus and crypt epithelial cells from the acutely denervated jejunum but not innervated controls [87]. Phorbol myristate acetate (PMA), a protein kinase C (PKC) activator, has no effect on ClC-2 levels.

#### 6.1.7. Dynein Motor Complex

Dhani and colleagues showed that expression of ClC-2 at the cell surface may be regulated via an interaction with the dynein motor complex [88]. The dynein intermediate chain co-immunoprecipitates with ClC-2 from hippocampal membranes, suggesting that they also interact *in vivo*. Disruption of dynein motor function perturbs ClC-2 localization and increases the functional expression of ClC-2 in the plasma membranes of COS7 cells. Thus, cell surface expression of ClC-2 may be regulated by dynein motor activity.

### 6.2. Functional Regulation

In addition to expression regulation, ClC-2 function is also susceptible to modulations by activation of many signaling mediators and by microenvironment changes induced by metabolic substances. This complex network is composed of a wide spectrum of factors that work cooperatively to define the function of ClC-2 channel proteins.

#### 6.2.1. Acidification

Under many circumstances, cellular (both extra- and intracellular) acidification occurs. It has been mentioned that ClC-2 channel activities are sensitive to pH changes. Acidification exerts dual effects on ClC-2 function: moderate extracellular acidification activates ClC-2, whereas strong acidification inhibits it [17,37,67,69,89–91]. An extracellular facing histidine, H532, was identified as a main structural determinant for channel closure when protonated [17]. The H532F mutation abolishes acidification-dependent channel inhibition, and the voltage-dependence of activation is imparted by voltage-dependent protonation of the gating glutamate [17].

#### 6.2.2. Protein Kinase A (PKA)

Several pharmacological and molecular studies suggest that ClC-2 may be regulated by protein phosphorylation events [6,91–96]. In the presence of intracellular PKA, an inwardly rectifying Cl^−^ current can be elicited by a hyperpolarizing voltage at a potential more negative than −50 mV. This same current is also activated by extracellular vasoactive intestinal peptide, and it can be suppressed by the PKA inhibitor H-89 [95].

In HEK-293 cells stably expressing human recombinant ClC-2 cDNA, the ClC-2 current is activated by cAMP-dependent PKA via a combination of forskolin plus IBMX but is inhibited by the cell-permeable myristoylated PKA inhibitor (mPKI) [91]. Moreover, low pH-induced channel activation is increased by PKA and prevented by mPKI.

In a subsequent study by [96], the PKA phosphorylation site(s) in ClC-2 protein were investigated by point mutations of consensus phosphorylation sites. The double phosphorylation site (RRAT655A plus RGET691A) mutation abolishes ClC-2 activation by PKA and low pH. Either the RRAT or RGET site is sufficient for PKA activation of ClC-2. Low pH activation of ClC-2 activity is PKA-dependent, retained in the RRAT655A mutant, but lost in the RRAT655A mutant. The RRAT655D mutant is constitutively active and can be further activated by PKA. These results show that activation of ClC-2 is differentially regulated by PKA at two sites, RRAT655 and RGET691 [96].

However, controversy exists as to the functional outcomes of PKA phosphorylation of ClC-2 channels. It has been reported that protein kinase A-dependent phosphorylation of ClC-2 fails to regulate either the magnitude or the kinetics of the hyperpolarization-activated Cl^−^ currents [97]. *In vivo* and *in vitro* phosphorylation of ClC-2 by PKA activation has been confirmed. In this study, phosphorylation of ClC-2 by PKC and Ca^2+^/calmodulin-dependent protein kinase II was excluded.

#### 6.2.3. Epidermal Growth Factor Receptor (EGFR)

Several reports point to a link between protein tyrosine phosphorylation and activation of ClC-2-like channel currents [98–100]: epidermal growth factor receptor (EGFR) potentiates the hypotonicity-induced anionic efflux. EGFR can be activated by several ligands, but TGF-α is thought to be the most physiological ligand for EGFR in the nervous system and the gastrointestinal tract because of its abundance in these tissues. Transforming growth factor-alpha (TGF-α) was found to irreversibly inhibit ClC-2 current in nystatin-perforated whole cell patch-clamp experiments in human colonic epithelial (T84) cells [101]. This effect requires activation of EGFR tyrosine kinase activity, phosphoinositide 3-kinase, and protein kinase C.

#### 6.2.4. Arachidonic Acid and Amidation

The whole-cell current generated by recombinant ClC-2 in HEK-293 cells can be activated by low concentrations of arachidonic acid [24,91], and single-channel activity of recombinant ClC-2 is activated by amidation [24,37]. Consistently, native ClC-2 channel activities are also activated by these agents. Arachidonic acid activation of ClC-2 channels is not inhibited by the PKA or PKC inhibitors mPKI or staurosporine; therefore, it is likely independent of PKA or PKC activation. The amidation activation of ClC-2 appears to result from one or more carboxyl groups that are available on the outer surface of the channel. Amidation removes tonic inhibition at neutral pH [37].

#### 6.2.5. Heat-Shock Proteins

Hsp70 is known to play an important role in protein folding, quality control, and membrane translocation processes [102]. Hsp90, another heat shock protein, is thought to control the conformational maturation and protein activities within multichaperone complexes containing Hsp70 [103]. These proteins are activated in response to cellular stresses such as elevated temperature, ischemia, or oxidative reagents. Both Hsp70 and Hsp90 can be co-immunoprecipitated with ClC-2 protein stably expressed in HEK293 cells [104]. Inhibition of Hsp90 by a specific inhibitor, geldanamycin or radicicol, does not affect total amounts of ClC-2 but reduces plasma membrane channel abundance. Whole cell patch-clamp recording showed that inhibition of Hsp90 reduces the ClC-2 current amplitude and slows the activation kinetics in a Cl^−^-dependent manner. Heat shock treatment produces the opposite effect. These results indicate that association of Hsp90 with ClC-2 results in greater channel activity due to facilitation of channel opening and enhanced channel sensitivity to intracellular [Cl^−^].

#### 6.2.6. Actin Cytoskeleton

An interesting study described that in the *Xenopus* oocyte expression system, the channel activity of ClC-2 is enhanced after treatment with the actin-disrupting agents cytochalasin and latrunkulin [105], indicating that the actin cytoskeleton normally exerts an inhibitory effect on ClC-2 activity. A glutathione S-transferase fusion protein containing the inhibitory domain in the *N*-terminus of ClC-2 is capable of binding actin in overlay and co-sedimentation assays. Furthermore, the binding of actin to S-transferase might be mediated through electrostatic interactions because binding is inhibited at high NaCl concentrations, with a half-maximal decrease in signal at 180 mM NaCl.

#### 6.2.7. Intracellular ATP

Adenosine triphosphate (ATP) and other nucleotides have been shown to bind to *C*-terminal cystathionine-β-synthase (CBS) domains of ClC-2. Using whole-cell patch clamp recordings, Stϕlting *et al.* demonstrated that ATP slowed down macroscopic activation and deactivation time courses in a dose-dependent manner [19]. Removal of the complete C-terminus abolished the effect of ATP, suggesting that CBS domains are necessary for ATP regulation of ClC-2 gating. Single-channel recordings identified long-lasting closed states of ATP-bound channels as the basis of this gating deceleration. Intriguingly, ClC-2 channels carrying naturally occurring sequence variants (G715E, R577Q and R653T) have been found in patients with idiopathic generalized epilepsy, and these variants accelerate common gating in the presence but not in the absence of ATP [19].

#### 6.2.8. Membrane Cholesterol Content

Apart from extra- and intracellular factors, the membrane lipid environment also can have a significant impact on ClC-2 function. In a specific study on this issue, detergent-resistant and detergent-soluble microdomains (DSMs) were isolated from stably transfected HEK293 cells [106]. ClC-2 was found to be concentrated in detergent-insoluble membranes under basal conditions and relocalized to DSMs upon cholesterol depletion by methyl-β-cyclodextrin, which was accompanied by an acceleration of the channel activation kinetics. Cells treated with the oxidant tert-butyl hydroperoxide and after ATP depletion demonstrated a similar distribution and activation pattern. In both cases, ClC-2 activation was prevented by cholesterol enrichment of cells. Thus, the cholesterol environment regulates ClC-2 activity, and the increase in ClC-2 activity in response to acute oxidative or metabolic stress involves relocalization of this channel to DSMs.

## 7. ClC-2 Channelopathies

ClC-2 has been generally believed to be associated with some human diseases when ClC-2 expression and/or function is disturbed. However, while the available research data indeed support some of the claims, they also disprove others.

### 7.1. Eye Disease

#### 7.1.1. Retinal Degeneration

Experimentally, ClC-2-deficient mice suffer from progressive retinal degeneration because of the early-onset loss of retinal photoreceptors [39]. These researchers observed severe retinal degeneration that eventually led to complete loss of photoreceptor cells. The onset of cell loss coincides with the formation of the blood–organ barrier by the retinal pigment epithelium (RPE) in the eye. The RPE has close spatial and functional relationships with photoreceptors, and transports fluid and lactate [107,108]. It is involved in phagocytosis of outer segments that are shed from photoreceptors, and synthesizes and transports metabolites (e.g., retinoids). The researchers tested their hypothesis that ClC-2 is similarly involved in the ionic homeostasis of the narrow subretinal space that is formed between the RPE and photoreceptors. ClC-2 is not only expressed in the neuronal retina but also in the RPE [55]. ClC-2 may play an important role in the regulation of pH in this compartment, or in the fluid transport across the RPE, which is important to prevent retinal detachment. This theory is compatible with the notion that photoreceptors degenerate as a consequence of an altered microenvironment created by the RPE.

Clinically, homozygous nmf240 mutants exhibit a grainy retina that progresses to panretinal patches of depigmentation. The mutation localized to a region on chromosome 16 containing the CLCN2 gene is associated with retinal degeneration [109]. Sequencing identified a mis-sense C-T mutation at nucleotide 1063 in CLCN2 that converts a glutamine to a stop codon. Mice homozygous for the CLCN2 (nmf240) mutation develop early onset and severe loss of photoreceptor cells at 14 days of age with only one layer of photoreceptor cells remaining at P21 that is preceded by elongation of the RPE apical microvilli. In nmf240 heterozygotes, the electroretinogram light peak, generated by the RPE, is reduced, though a normal retinal histology is present [109].

#### 7.1.2. Sjögren’s Syndrome

Sjögren’s syndrome is a systemic autoimmune disease, which causes functional impairment of the lacrimal and salivary glands that produce tears, and is one of the most common causes of dry eye [110]. Rabbits with induced autoimmune dacryoadenitis (IAD) mimic many of the ocular surface symptoms as well as lacrimal gland (LG) pathological features characteristic of Sjögren’s syndrome. Nandoskar *et al.* demonstrated that there were significant changes of mRNA and protein expressions of NKCC1, CFTR, and ClC2γ in rabbits with IAD [111]. Specifically, ClC2γ mRNA is markedly decreased in interlobular and interlobar ducts from rabbits with IAD, suggesting decreased secretion or increased absorption of ClC2γ-mediated Cl^−^ transport in these two duct segments. These data provide suggestive evidence for the potential contribution of ClC-2 to the altered Cl^−^ transport in rabbits with IAD; however, direct functional studies are needed to provide definite evidence.

### 7.2. Male Infertility

#### 7.2.1. Testicular Degeneration

In the testes, tight junctions between Sertoli cells effectively isolate the adluminal compartment of seminiferous tubules. Germ stem cells and spermatogonia are located on the cis (blood) side of the barrier, and the differentiation to sperm cells requires a tight physical association with Sertoli cells. The blood–testis barrier requires that Sertoli cells transport or synthesize many essential nutrients. Disruption of the ClC-2-encoding gene in mice leads to degeneration of male germ cells, resulting from a defect in transepithelial transport across Sertoli cells [39]. Testicular degeneration in ClC-2 knockout mice begins at ~2 weeks of age, concomitant with the establishment of the blood–testis barrier. The lack of lumina in seminiferous tubules suggests a defect in transepithelial transport by Sertoli cells, which express ClC-2 at their cell surface and whose morphology is abnormal in ClC-2 knockout mice.

#### 7.2.2. Azoospermia

Azoospermia, the condition that no measurable level of spermatozoa is present in semen and ejaculate fluids, is associated with male infertility. There are two clinically distinct forms of azoospermia; obstructive azoospermia occurs when spermatozoa are produced but cannot mix with seminal fluids due to a physical, obstructive barrier, and nonobstructive azoospermia arises from a disruption in spermatogenesis. In mice expressing a ClC-2nmf240 mutation, the loss-of-function ClC-2nmf240 mutant results in nonobstructive azoospermia: spermatogenesis is arrested, and atrophy of the testes begins as early as 6 weeks of age and is uniformly present by 20 weeks of age [109]. These phenotypes resemble those reported in genetically engineered homozygous ClC-2 knockout mice, despite the fact that the ClC-2nmf240 mutants appeared to have an earlier onset of infertility and azoospermia [109].

### 7.3. Chronic Constipation and Irritable Bowel Syndrome

Chronic constipation is a common health problem occurring in approximately 4.5 million Americans, affecting up to 27% of the population and negatively impacting health-related quality-of-life. Functional ClC-2 is expressed in both the colon and rectal surface epithelium. Several lines of evidence indicate that ClC-2 may participate in fluid secretion in the murine small intestine [112,113] or in fluid absorption in the colon, as suggested by immunolocalization [114,115] and functional data [116]. In colons of ClC-2 knockout mice, electroneutral Na^+^, K^+^, and Cl^−^ absorption is dramatically reduced. Basolateral ClC-2 channels are required for colonic electroneutral absorption of NaCl and KCl [117]. In the rectum, the channel activity may be negligible and thus nonessential for controlling electrogenic Na^+^ transport in this surface epithelium under basal physiological conditions [118]. The recent development of lubiprostone (Amitiza), a ClC-2 activator for the treatment of chronic constipation, may be the most solid and convincing evidence for the role of ClC-2 in chronic constipation. Lubiprostone is a bicyclic fatty acid that acts locally on ClC-2 channels located in the apical membrane of intestinal epithelial cells. Lubiprostone activates a Cl^−^ channel and increases Cl^−^ secretion. Preclinical trials have shown high specificity of the drug for ClC-2 channels. Stimulation of Cl^−^ secretion by lubiprostone induces the passive movement of Na^+^ and water into the intestinal lumen, yielding a net increase in isotonic fluid to improve bowel function, stool consistency, bloating, and global relief of constipation compared with placebo. In double-blind, placebo-controlled clinical trials, lubiprostone increased the number of spontaneous bowel movements compared with placebo and was generally well tolerated [27–29]. Lubiprostone has been evaluated in six placebo-controlled, double-blind, randomized Phase II or III clinical trials, and has been approved by the FDA for the treatment of chronic constipation in men and women and the treatment of women with irritable bowel syndrome with constipation.

### 7.4. Neuronal Disease

#### 7.4.1. Epilepsy

As already introduced in an earlier section, ClC-2 has been proposed to participate in lowering the cytoplasmic chloride concentration ([Cl^−^]_i_) of neurons so as to suppress neuronal excitability [60,62,119]. Though Cl^−^ extrusion is primarily mediated by K^+^/Cl^−^ cotransporter 2 (KCC2), additional mechanisms should strengthen the process, particularly under conditions of high intracellular Cl^−^ load. As ClC-2 has a large conductance, it is especially suited for this purpose [62]. Additionally, if ClC-2 conductance is active at the resting membrane potential, it must take part in controlling the membrane resistance [120]. Hence, loss of ClC-2 may enhance neuronal excitability to favor epilepsy, whereas activation of ClC-2 may suppress neuronal excitability by inducing an inhibitory response to GABA. In humans, mutations in the ClCN2 gene predict hyperexcitability of GABAergic synapses, which can lead to epilepsy. However, whether ClC-2 mutations indeed cause epilepsy or not has been controversial.

On one hand, based on studies performed with hippocampus cells [60] or transfected dorsal root ganglion neurons [62], ClC-2 has been implicated in the regulation of the effects of GABA_A_ receptor action by controlling intracellular Cl^−^ concentration. The GABR_A1_ gene was originally implicated in familial juvenile myoclonic epilepsy [121]. Moreover, ClC-2 is abundantly expressed in the brain, and the current consistently mediated by ClC-2 channels has been detected in glial cells and neurons. In theory, ClC-2 in certain neurons such as pyramidal cells could act to prevent the accumulation of intracellular Cl^−^ above electrochemical equilibrium to limit the excitation mediated by glycine or GABA_A_ receptors by preventing excessive Cl^−^ accumulation induced by activation of glycine and GABA_A_ receptors during high-frequency neuronal activity. Evidence for this notion came from experiments showing that introducing ClC-2 could confer GABA-dependent inhibition to dorsal root ganglion neurons showing GABA-evoked excitation [62]. A recent study showed that ClC-2 mediates the chloride current, is involved in chloride extrusion, and constitutes a substantial part of the background conductance in hippocampal neurons [63]. The loss of ClC-2 leads to a dramatic increase of the input resistance of CA1 pyramidal cells, thereby increasing excitability. Surprisingly, basal synaptic transmission decreased in field recordings, which was dependent on GABAergic inhibition. A subset of interneurons displays a characteristic ClC-2-mediated current, and the loss of ClC-2 in interneurons leads to an increase of excitability of interneurons, resulting in an increased inhibition of principal neurons, thereby reducing the overall network excitability [63]. A more recent study claimed that ClC-2 is functionally upregulated in CA1 pyramidal cells in pilocarpine-treated rats, which is reversed by L655,708, a specific antagonist of the α5 subunit-containing GABA_A_ receptor [122]. The findings suggest that ClC-2 contributes to tonic inhibition mediated by the α5 subunit-containing GABA_A_ receptor in the CA1 region in experimental rats with temporal lobe epilepsy. Finally, mutations in the CLCN2 gene encoding ClC-2 in humans have been linked to epilepsy [123,124].

A recent study by Klassen *et al.* reveals the complex variant profiles of ion channel genes in idiopathic epilepsy (IPE), and they found that the large single-nucleotide polymorphisms (SNPs) of several ion channel genes are observed in controls, and most SNPs are shared between controls and IPE patients, suggesting that disease-causing SNPs are possibly masked in the controls by other SNPs [125]. Since several ion channel gene products contribute to increased neuronal excitability in IPE, small alterations of several ion channels might cooperatively change neuronal excitability. Therefore, SNPs are expected not to produce pronounced effects on channel gating for altering cell excitability [125]. However, Stolting *et al.* recently identified that several disease-associated ClC-2 mutants exhibit a common gating alteration, suggesting that changes in ClC-2 gating may contribute to cell excitability [19].

On the other hand, over the past several years, several mutations in CLCN2 have been identified [123,124,126–128], three of which were believed to be co-segregated in an autosomal-dominant fashion with idiopathic generalized epilepsies in the study reported by Haug *et al.* in 2003 [35]. However, the epilepsy-related pathology of two mutations, which are supposed to cause severe truncation of ClC-2 protein and altered splicing with nearly complete loss of a large transmembrane helix and were predicted to cause dominant negative effects, has not been confirmed by others [2,129]. In fact, the 2003 study by Haug *et al.* was later retracted by the authors [130]. Further negative evidence came from the possibility that ClC-2 as a single epilepsy-causing gene is excluded as ClC-2 mutations in humans were not found to cause impressive changes in the biophysical properties of ClC-2 [131]. Therefore, the ClC-2 sequence abnormalities previously found in patients with epilepsy most likely represent innocuous polymorphisms. Additionally, increased susceptibility to seizures was not observed in mice with complete disruption of ClC-2, and neurological deficits were found to be mainly mild with decreased conduction velocity in neurons of the central auditory pathway [39,42,131]. These results all seem to disfavor epilepsy as a ClC-2 channelopathy. In addition, it remains questionable as to whether the Cl^−^ efflux via ClC-2 can lead to sufficient GABAergic inhibition under prolonged paroxysmal conditions that could adequately limit neuronal hyperexcitability.

The absence of an overt seizure-susceptibility phenotype in young ClC-2 knockout mice was initially described by Bösl *et al.* and subsequently reported by Blanz *et al.* and Nehrke *et al.* [39,42,131]. In addition, studies on older ClC-2-deficient mice (>6 months) were conducted. The results revealed abnormalities in the myelin of central axons and a subtle defect in the neuronal function in the central auditory pathway. Electrocorticographic recordings showed spontaneous interictal spikes, which are a marker of perturbed hippocampal neurotransmission with a resultant increase in excitation. This electrophysiological defect was found to be associated with astrocyte activation and neuronal degeneration in the CA3 region of the hippocampus of these older mice. The authors suggest that ClC-2 expression plays a subtle neuroprotective role in the aging hippocampus [132].

#### 7.4.2. Leukoencephalopathy

Mutations in the gene MLC1 are found in approximately 80% of patients with the inherited childhood white matter disorder megalencephalic leukoencephalopathy with subcortical cysts (MLC), which is a leukodystrophy that is caused by mutations in MLC1 or GLIALCAM. An important phenotype in humans with MLC is vacuolation. A brain biopsy from an MLC patient revealed myelin [133] and astrocyte vacuolation [134]. It was suggested that MLC may be caused by impaired ion transport across cellular membranes, thereby leading to an osmotic imbalance and disturbed fluid homeostasis [134,135]. Similar to the case of epilepsy, whether ClC-2 plays a role in leukoencephalopathy is debatable. In mice, it was found that the disruption of ClC-2 causes fluid accumulation leading to myelin vacuolation [136]. ClC-2 was identified as a GlialCAM binding partner: GlialCAM and ClC-2 colocalize in Bergmann glia, in astrocyte-astrocyte junctions at astrocytic endfeet around blood vessels, and in myelinated fiber tracts. GlialCAM targets ClC-2 to cell junctions, increases ClC-2 currents, and changes its biophysical properties. Disease-causing GLIALCAM mutations abolish the targeting of the channel to cell junctions. This work suggests that ClC-2 may play a role in the pathology of MLC disease [136]. Similarly, early work demonstrated that a homozygous ClC-2 mutation at nucleotide 1063 in CLCN2, which converts a glutamine to a stop codon, experiences leukoencephalopathy in multiple brain areas [109].

However, a conflicting finding had been published earlier [137]. Mice lacking ClC-2 protein develop white matter abnormalities, which are characterized by vacuole formation in the myelin sheaths, strikingly similar to the intramyelinic vacuoles in MLC. Sequence analysis of CLCN2 at the genomic DNA and cDNA levels in 18 MLC patients without MLC1 mutations revealed some nucleotide changes, but these changes were predicted to be nonpathogenic. Furthermore, in electrophysiological experiments, one of the observed amino acid changes was found to have no effect on the ClC-2-mediated currents. These authors concluded that ClC-2 is not involved in MLC [137].

### 7.5. Cystic Fibrosis Disease

Cystic fibrosis (CF), also known as mucoviscidosis, is a chronic, progressive, autosomal, recessive, and frequently fatal genetic disorder that primarily affects the body’s mucus glands and critically alters the lungs as well as the pancreas, liver, and intestine in children and young adults. It is characterized by abnormal transport of Cl^−^ and Na^+^ across an epithelium, leading to thick, viscous secretions [138]. Mutations in the cystic fibrosis transmembrane regulator (CFTR) lead to death from lung disease. ClC-2 is expressed in epithelial cells throughout the respiratory tract, and activation of ClC-2 in the respiratory epithelia is a potential treatment for CF [10,24,67,89,90,139]. Indeed, it has been shown that ClC-2 channel expression in epithelial cells from CF patients can correct the defect in Cl^−^ transport *in vitro* [67]. In addition, keratinocyte growth factor (KGF) has been shown to cause CFTR-independent changes in lung morphogenesis *in vivo* and to raise the levels of ClC-2 channel protein in mouse lung explants *in vitro* [75].

However, presently, the role of ClC-2 in CF is apparently another controversial issue yet to be resolved. In the study by Haug *et al.* [35], mutations in the ClC-2 coding region fail to cause any lung disease, indicating that ClC-2 is not critical for the function of the mature respiratory epithelium when CFTR is present. Similarly, ClC-2 knockout mice have severe degeneration of the retina and testes, but no evident lung disease [39,42]. Several polymorphisms of key regulatory domains of the CLCN2 gene have been identified in a cohort of subjects with CF who carry the same CF genotype; however, no significant association of ClC-2 polymorphisms with CF argues against ClC-2 as a key modifier gene of the CF lung phenotype in adulthood [140].

### 7.6. Cardiovascular Disease

There are several reasons to believe that ClC-2 should play a critical role in cardiac electrophysiology and pathophysiology, in view of the biophysical characteristics of the ClC-2 channel and its expression in cardiac myocytes. First, during a cardiac action, potential, activation of ClC-2 channels primarily conducts an inward current with Cl^−^ efflux at membrane potentials negative to the Cl^−^ equilibrium potential (approximately −30 mV). In this way, ClC-2 expectedly serves to depolarize the membrane of cardiac cells. Second, at membrane potentials more positive than the Cl^−^ equilibrium potential, ClC-2 mediates an outward current as a result of Cl^−^ influx and thus tends to accelerate the rate of membrane repolarization and shortens the action potential duration. Third, ClC-2 can aid spontaneous diastolic membrane depolarization and pacemaker activities because of the inward current it carries during hyperpolarization and its distribution in sino-atrial nodal cells that resemble the distribution and function of the cationic pacemaker channels (*I*_f_). Fourth, despite that ClC-2 conductance may be small under isotonic conditions, it can be augmented by acidosis [141] and hypotonic cell swelling [15], conditions manifested in myocardial ischemia and cardiac hypertrophy. Under such conditions, the significance of ClC-2 in the heart is deemed to become more prominent, and there is also a possibility that ClC-2 participates in these pathological processes, particularly the associated arrhythmogenesis as a result of abnormal membrane depolarization, enhanced automaticity generating ectopic excitations, or excessive shortening of the action potential duration occurring in acute myocardial ischemia. Nevertheless, no research has thus far been published to support these views, and the role of ClC-2 in the heart pathophysiology remains purely speculative.

## 8. Conclusions and Perspectives

After years of intensive and extensive experimental and clinical investigations, there is no doubt that we have come to a stage of a much broader and deeper understanding of ClC-2 channels in terms of the biophysical characteristics, pharmacological properties, molecular features, cellular functions, regulatory mechanisms, and pathophysiological implications. In particular, several ClC-2 channelopathies have been well established by solid experimental findings and clinical observations, such as retinal degeneration, testicular degeneration, azoospermia, chronic constipation, and irritable bowel syndrome. Nonetheless, we have to admit that our current knowledge regarding ClC-2 is still far from complete. Especially, the links between biophysical properties and cellular functions, and links between cellular functions and pathological roles have not been firmly established and even have been contradictory. Many aspects of ClC-2 physiology and the underlying mechanisms remain uncertain and even mysterious. These notions are particularly reflected by the very limited overt phenotypes in ClC-2-deficient mice [39,42,131] and in ClC-2 mutations in humans [2,129]. Several possibilities may explain the discrepancies between the anticipated pathological roles based on the electrophysiology and the actual phenotypes.

1. Expression levels of ClC-2 in certain tissues are not sufficiently high to give rise to phenotypes. For example, six distinct types of sarcolemmal Cl^−^ currents coexist in cardiac myocytes, and ClC-2 is one of them [16,142–145]. The expression level of ClC-2 in the heart is significantly less than that of ClC-3 [146]. Furthermore, the functional activity elicited by the molecular counterpart (ClC-2 current) exits only in a small population of cardiac myocytes, which is no match with the expression level of ClC-2 [15].
2. It may be that ClC-2 plays a pathophysiological role in many tissues, but overt phenotypes are restricted to only a limited number of tissues where the loss of its effects cannot be compensated by other proteins. Again, taking the heart as an example, ClC-2 and ClC-3 were found to be co-localized in sarcolemmal membranes of cardiac cells [15], and impairment of ClC-2 may well be compensated by ClC-3 for some of the cellular functions that they share.
3. The third possibility is that there are species differences between rodents and humans in terms of ClC-2 pathophysiology, and the results acquired from mouse models may not be extrapolated to humans.

Future studies on ClC-2 will be continuously directed to identify the pathophysiological roles or the relationships of this channel with human disease. While it is unlikely that rodent models can further advance our understanding, alternative approaches are definitely needed: but what? The development of the specific intestinal ClC-2 activator lubiprostone for the treatment of chronic constipation gives us a hint. The efficacy of lubiprostone not only provides a new therapy but also verifies the pathophysiological function of ClC-2. In other words, functional exploitation based upon biophysical characteristics may not lead to any further insights; instead, a pharmacological approach based upon the pharmacological properties of ClC-2 may yield more fruitful and reliable results. The effects of this compound in other tissues/organs should be investigated to broaden our view of the function of ClC-2. In addition, specific ClC-2 inhibitors/blockers need to be developed to study the pathophysiological roles of ClC-2.
